# Editorial: How does sleep help regulate negative emotion?

**DOI:** 10.3389/fnbeh.2022.1125841

**Published:** 2023-01-09

**Authors:** Edward F. Pace-Schott, Birgit Kleim, Candice A. Alfano

**Affiliations:** ^1^Department of Psychiatry, Harvard Medical School, Massachusetts General Hospital, Boston, MA, United States; ^2^Department of Psychology, University of Zurich, Zurich, Switzerland; ^3^Department of Psychology, University of Houston, Houston, TX, United States

**Keywords:** emotion regulation, sleep, memory, dreaming, psychopathology

## Introduction

In this volume, 15 scientific reports give a snapshot of some of the most exciting current research on how sleep helps regulate negative emotion. Articles illustrate state-of-the-art research paradigms from the cognitive, affective, and behavioral neurosciences, sleep medicine, biological psychiatry, and dream research.

## Review and meta-analysis

This collection begins with a narrative review of sleep and emotional memory by Cunningham et al. and a meta-analysis of sleep and emotional reactivity by Lipinska et al.. Cunningham et al. focus on the timing of sleep manipulations and assessments in relation to memory encoding. They note that inconsistent findings in the literature may result when different memory processes occurring at different times following encoding confound one another. Both studies emphasize the limitations of empirical findings to date and advocate for increased sophistication, rigor and statistical power in sleep science. Specifically, they note that two tenets of conventional wisdom in sleep science—that sleep preferentially consolidates emotional memories while serving to moderate emotional reactivity—are generally unsupported or supported only under certain conditions.

Nonetheless, both reviews identify certain general findings. Cunningham et al. note that extreme sleep deprivation imposed at all stages of information processing negatively impacts memory, irrespective of whether or not memory holds emotional content (see also Davidson et al., 2021). Similarly, they report that total sleep deprivation (TSD) prior to encoding impairs memory of both emotional and neutral information equally, with the possible exception of aversive memories. Lipinska et al. report overall increases in subjective arousal ratings to negative stimuli following sleep whereas, following TSD, positive stimuli evoke more negative responses. Lipinska et al. also note the dearth of studies employing simple and affordable psychophysiological measures of arousal.

## Sleep deprivation studies

Groeger et al. compared responses on the Positive and Negative Affect Scale (PANAS) after 40 h TSD following either a week of sleep-restriction (SR) or sleep-satiated laboratory sleep. They additionally examined changes in PANAS across the course of a forced desynchrony (FD) protocol. Interestingly, they showed larger effect sizes on positive vs. negative mood scales of the PANAS for all three of these sleep manipulations.

Kurinec et al. examined whether changes in electrodermal activity (EDA) during TSD compared to normal sleep correspond to changes in sleepiness, vigilant attention, and affect in a large sample of healthy adults. Skin conductance level (SCL) and rate of spontaneous skin conductance responses (NSSCR) were obtained over a 5-min rest interval. TSD produced expected changes in positive affect, sleepiness, and vigilance, while NSSCR but not SCL decreased following TSD. Neither parameter, however, correlated with any other measurements, suggesting that EDA does not adequately reflect experiences of arousal during either rested wakefulness or TSD.

Sundelin and Holding tracked state anxiety (Spielberger State anxiety index; STAI-S) across two large samples of healthy young adults, one of which underwent a full night's TSD while the other underwent two continuous days of sleep restricted to 4 h. In both, sleep loss resulted in an increase in state anxiety which correlated with trait anxiety (STAI-T). However, trait anxiety did not moderate the relationship between sleep loss and state anxiety suggesting that those with high levels of trait anxiety do not show a proportionately greater effect of sleep loss on state anxiety. These results notably conflict with findings from other published studies (Palmer and Alfano, 2020), highlighting a need for greater investigation of the potential impact of trait anxiety on emotional responses following sleep loss.

Thompson et al. examined interrelationships of inflammatory cytokines and stress hormones with cognition and mood across 24 h of TSD compared to baseline in healthy young adults. TSD resulted in an increase of inflammatory markers (CRP and IL-6) and suppressed the morning cortisol peak. In addition, they found TSD increased reaction time (RT) on some cognitive tasks (without impairing accuracy) and increased negative mood states (Profile of Mood States; POMS) and anxiety (STAI-S).

## Targeted memory reactivation

Borghese et al. and Halonen et al. applied targeted memory reactivation (TMR), a cutting-edge experimental paradigm in sleep science (Schouten et al., 2016; Lewis and Bendor, 2019; Hu et al., 2020), to emotional memory. In TMR, specific sensory stimuli (e.g., odor, sounds) are associated with a learning session after which the associated stimulus and/or a control stimulus are presented during sleep. Memory performance is then compared, within- or between-subjects, after waking between sleep presentation of the learning-associated stimulus and its control and is often found superior following the associated stimulus (Hu et al., 2020). The stimuli are typically presented during slow wave sleep (SWS) during which the influential active systems consolidation model (Klinzing et al., 2019) suggests that coupling of specific EEG oscillations facilitate memory consolidation. However, the association of REM sleep with emotion processing has prompted these investigators [and a few others (Hutchison et al., 2021)] to examine the possible use of TMR during REM.

Halonen et al. used an embarrassment-induction procedure (Wassing et al., 2019) with replay of a sound stimulus or sham stimulus during REM or SWS. When tested for overnight habituation using skin-conductance response (SCR), they found no effect of TMR during either stage. However, greater proportion and fragmentation of REM was negatively associated with habituation and this effect was moderated by trait shame proneness.

Borghese et al. similarly used one week of acoustic TMR during REM sleep in persons with social anxiety disorder treated with two virtual reality exposure sessions involving public speaking. The encoding experience paired with replayed stimuli was the positive feedback given to participants after each exposure. Although TMR itself had no effect, having more REM sleep was associated with less anxiety (increased parasympathetic tone) when preparing for a subsequent exposure.

These results concur in finding no effect of TMR during REM (Hu et al., 2020). However, in one case, greater REM was associated with greater anxiety whereas in the other it was associated with less.

## Applications to psychiatry

### Blue light

In addition to the well-known circadian entrainment function of intrinsically photosensitive retinal ganglion cells, photostimulation of this pathway also affects mood, alertness and cognition (Chellappa et al., 2011; Lazzerini Ospri et al., 2017; Fernandez et al., 2018). Killgore, Vanuk et al. and Vanuk et al. examined the effects of morning blue light treatment compared to an amber light control given over 6-weeks in patients with post-traumatic stress disorder (PTSD). Blue light treatment lengthened sleep relative to the amber control whereas the amber light improved sleep efficiency (Killgore, Vanuk et al.). Structural MRI showed increased amygdala volume in the blue-light group that correlated with decreased nightmare severity. Vanuk et al. showed that the blue light treatment was superior in reducing participants' sleep complaints. Although both treatments reduced the severity of PTSD symptoms, only in the blue light group did these improvements correlate with improved sleep. Additionally, the blue group better maintained extinction learning at 6 weeks compared to the amber group.

### Suicidal ideation

Killgore, Grandner et al. report that the personality trait of extroversion is associated with elevated suicidal ideation in sleep deprived individuals and in those with insomnia. This was seen in both military service members in an extreme sleep deprivation protocol and among those with clinical levels of insomnia in a large community sample. Persons higher on the extroversion scale are more vulnerable to both the subjective and objective effects of sleep deprivation (Killgore et al., 2007). They hypothesize that this extroversion-associated risk may result from the synergistic effects of inattention and impulsivity, the former resulting from sleep deprivation and the latter being positively correlated with trait extroversion.

Similarly, Tavakoli et al. found that, in adolescents who had been hospitalized for a suicide attempt, performance on a test of inhibitory control negatively correlated with REM pressure (latency and percent) and sleep continuity. In contrast, an evoked response potential (ERP) index of inhibitory processing was positively correlated with SWS. The authors point out that this pattern mirrors the REM disinhibition and decreased SWS that is associated with adult depression.

### Mindfulness

Mamede et al. used structural equation modeling (SEM) of data from a large online questionnaire study to examine interrelationships of sleep and trait mindfulness among other factors in explaining depressive and anxiety symptoms during the COVID-19 pandemic. They found significant mediation of the effects of mindfulness on overall mental health by the combined effects of rumination and insomnia symptoms.

## Emotion regulation during dreaming

Barbeau et al. report evidence for an emotion regulatory function of dreams by comparing emotionality reported by the dreamer to emotionality rated by judges. They describe lesser emotionality in dream self-reports as a “positivity bias”, quantified by subtracting the dreamers' mood ratings from judges' ratings. These authors found positivity bias to predict degree of positive mood reported upon waking.

In contrast, Sikka et al. provide evidence for a lack of an emotion regulatory effect of dreaming. Beginning with the common hypothesis that negative emotion in dreams functions to discharge or “work-through” negative emotion in wakefulness, these investigators tested whether, following nights containing negatively-toned dreams, individuals were less reactive to negatively-toned images and were better able to down-regulate negative responses *via* cognitive reappraisal. However, they found just the opposite: more negatively-toned dreams were associated with more negatively-toned post-sleep mood and affected neither the reactivity to negative images nor the ability to re-appraise. They postulate their findings support a continuity between dream and waking mood, as would be predicted by the “continuity hypothesis” (Schredl and Hofmann, 2003).

## Conclusions

The mixed findings in this volume are instructive in illustrating how differences in experimental paradigms and measures can lead to quite different conclusions. Such differences aside, findings indicate that sleep is indeed an important component of emotion regulation, and that the specific mechanisms are fertile ground for future research.

## Author contributions

All authors listed have made a substantial, direct, and intellectual contribution to the work and approved it for publication.
